# Incidence of pneumonitis/interstitial lung disease induced by HER2-targeting therapy for HER2-positive metastatic breast cancer

**DOI:** 10.1007/s10549-020-05754-8

**Published:** 2020-06-26

**Authors:** Michelle D. Hackshaw, Heather E. Danysh, Jasmeet Singh, Mary E. Ritchey, Amy Ladner, Corina Taitt, D. Ross Camidge, Hiroji Iwata, Charles A. Powell

**Affiliations:** 1 Daiichi Sankyo, Inc., 211 Mount Airy, 1A-453, Basking Ridge, NJ 07920 USA; 2 RTI Health Solutions, Waltham, MA USA; 3 RTI Health Solutions, Research Triangle Park, NC USA; 4 University Colorado, Aurora, CO USA; 5 Aichi Cancer Center, Nagoya, Japan; 6 Mount Sinai Hospital, New York, NY USA

**Keywords:** Metastatic breast cancer, HER2 positive, Interstitial lung disease, HER2-targeting therapy, Trastuzumab, Lapatinib, Trastuzumab emtansine, Trastuzumab deruxtecan, Trastuzumab duocarmazine

## Abstract

**Purpose:**

Anti-human epidermal growth factor receptor 2 (HER2) therapies are associated with interstitial lung disease (ILD), also referred to as pneumonitis. In this literature review, we describe the incidence of ILD among patients with HER2-positive metastatic breast cancer (MBC) receiving anti-HER2 therapies, and we describe existing recommendations for monitoring and managing drug-induced ILD among these patients.

**Methods:**

We searched PubMed and Embase to identify clinical trials and postmarket observational studies that investigated anti-HER2 therapies for HER2-positive MBC, reported on ILD, and were published during January 1, 2009 to July 15, 2019. Articles were screened by two researchers; data were extracted from the full-text articles.

**Results:**

The 18 articles selected for this review assessed 9,886 patients who received trastuzumab (8 articles), lapatinib (4 articles), trastuzumab emtansine (3 articles), trastuzumab deruxtecan (2 articles), or trastuzumab duocarmazine (1 article). The overall incidence of all-grade ILD was 2.4% (*n* = 234), with 66.7% (*n* = 156) occurring as grade 1–2 events, 0.5% grade 3–4 (*n* = 54; incidence), and 0.2% grade 5 (*n* = 16; incidence). The highest ILD incidence (21.4%) was among patients receiving trastuzumab combined with everolimus and paclitaxel. Ten studies indicated that ILD events were managed via dose interruption, dose reduction, or treatment discontinuation; two studies included detailed guidelines on managing drug-induced ILD.

**Conclusions:**

ILD is a well-described adverse drug reaction associated with several anti-HER2 drugs. Published ILD management guidelines are available for few anti-HER2 treatment regimens; however, guidance for monitoring for anti-HER2 drug-induced ILD is lacking.

**Electronic supplementary material:**

The online version of this article (10.1007/s10549-020-05754-8) contains supplementary material, which is available to authorized users.

## Introduction

Approximately 15–20% of patients with breast cancer have tumors that overexpress human epidermal growth factor receptor 2 (HER2), which are associated with an aggressive clinical phenotype and poor prognosis [1, 2]. The development and clinical integration of anti-HER2 therapies, including trastuzumab, pertuzumab, lapatinib, neratinib, and trastuzumab emtansine (T-DM1), has resulted in extended survival in patients with HER2-positive breast cancer [3–7]. Several anti-HER2 therapies, as well as other anticancer agents (e.g., cyclin-dependent kinase-4/-6 inhibitors, immune checkpoint inhibitors, mammalian target of rapamycin [mTOR] inhibitors), have been linked to an increased risk of drug-induced interstitial lung disease (ILD) [3, 4, 7–10]. Drug-induced ILD encompasses a group of serious, and sometimes life-threatening pulmonary conditions characterized by fibrosis and inflammation of the lung interstitium [11, 12].

Corticosteroids or oral prednisone are frequently used pharmacologic therapies for ILD that may effectively slow or reverse ILD disease progression [12]. While guidance is available on the diagnosis and treatment of drug-induced ILD [13], there is limited guidance on disease monitoring and management for patients with ILD induced by HER2-targeted therapies. This may be due to variability in the terminology used to describe ILD in the literature, as well as limited provider experience in routinely diagnosing and managing ILD [12, 14].

The objective of this review is to describe the incidence and severity of drug-induced ILD across currently approved and investigational anti-HER2 therapies in female patients with metastatic breast cancer (MBC), and describe recommendations for monitoring and management of anti-HER2 drug-induced ILD among MBC patients.

## Methods

PubMed and Embase databases were searched for phase 2, 3, and 4 clinical studies that assessed current and investigational therapies for HER2-positive MBC. The predefined search terms included combinations of free text and Medical Subject Headings (MeSH). All databases were searched for English-language articles only, during the period of January 1, 2009, through July 15, 2019, and with no geographic restrictions imposed. Bibliographies of systematic literature reviews (SLRs) and recent articles published after the database search time window were examined to identify additional relevant publications.

Article screening and selection were conducted in two phases. In the first phase, titles and abstracts of articles identified from the electronic databases were reviewed by one researcher for eligibility according to the inclusion and exclusion criteria, which are outlined in Table S-1, Supplementary Material, including the terms used to describe ILD (i.e., pulmonary fibrosis, pneumonitis, organizing pneumonia, acute interstitial pneumonitis, diffuse parenchymal lung disease, pulmonary eosinophilia, ILD). In the second phase, the full text of the articles selected in the first phase was obtained and reviewed for eligibility by a second researcher using the same inclusion and exclusion criteria. Uncertainty about the inclusion of articles was resolved by discussion and consensus of two researchers. Information was extracted from full-text publications using a data extraction template with prespecified fields. Quality control procedures included verification of all extracted data with original sources by a researcher who did not perform the primary data extraction.

## Results

**Fig. 1:**
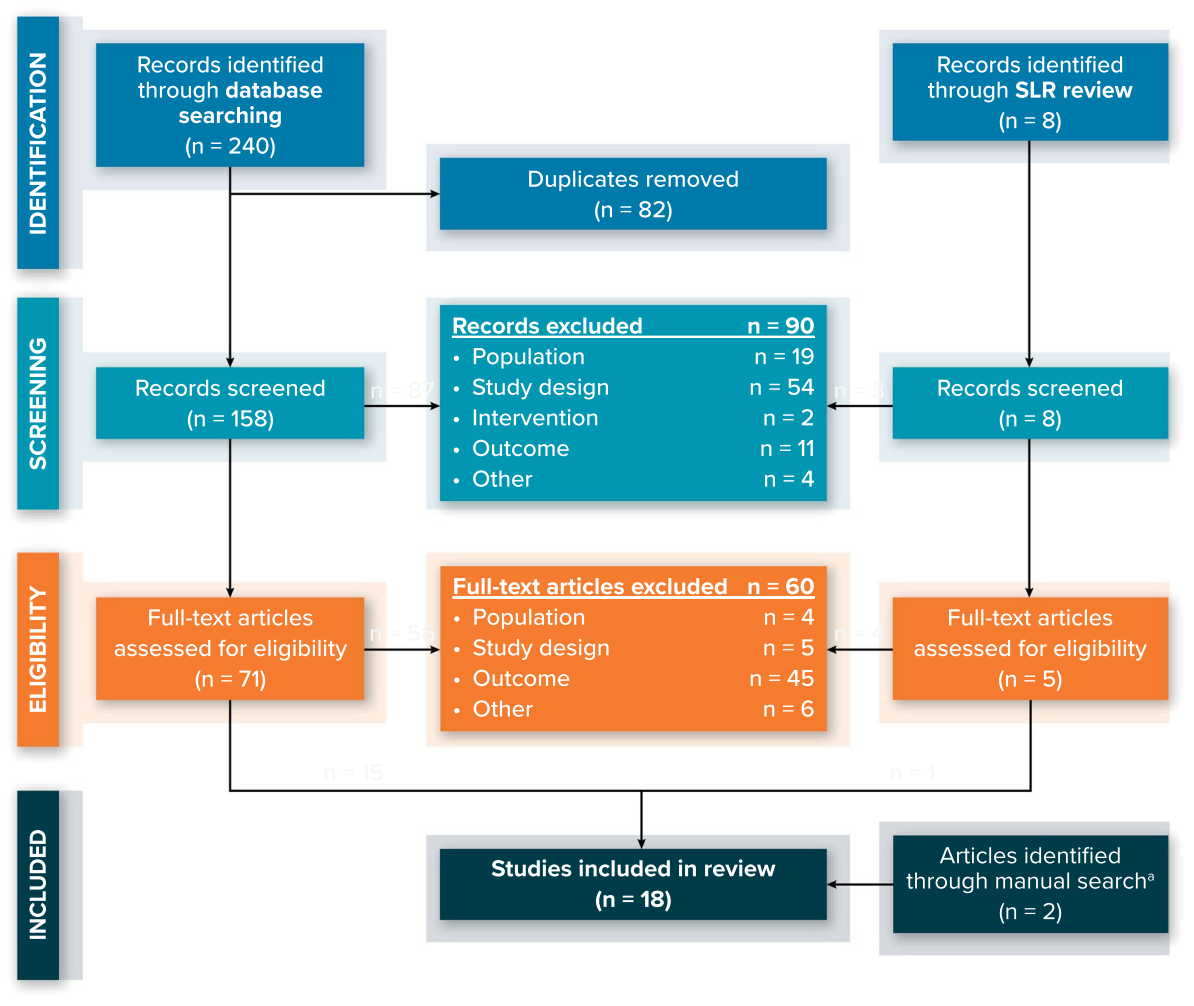
PRISMA Diagram of Articles Included in Review. *HER2* human epidermal growth factor receptor 2, *PRISMA* preferred reporting items for systematic reviews and meta-analyses, *SLR* systematic literature review. ^a^Includes one article on T-DXd published after the search cut-off date [15], and one phase 1 study describing trastuzumab duocarmazine, an investigational anti-HER2 drug that is suspected to be associated with treatment-related interstitial lung disease [16]

Of the 240 studies identified through database searching, 158 titles and abstracts were identified in the PubMed and Embase databases after removing duplicate articles, as well as eight additional articles identified through the SLR bibliography search (Fig. 1). Of the 166 total articles that were reviewed in the first screening phase, 76 were eligible for a full-text review based on the inclusion and exclusion criteria. During the full-text review, a total of 60 articles were excluded, with the majority (*n* = 45 [75.0%]) excluded because they did not report on ILD. Fifteen articles from the electronic database search and one from the SLR bibliography search were eligible for inclusion in the review. Two additional relevant studies (one for trastuzumab deruxtecan [T-DXd] [15] and one for trastuzumab duocarmazine [16]) were identified via manual searches, yielding a total of 18 articles that were included in the review.

The study and patient characteristics for the 18 articles in the review are presented in Table 1. Studies reported at least 99% enrollment of female patients, except for one study that reported enrolling 77% and 82% female patients in two study cohorts [16]. Age (median) at enrollment ranged from 47 to 57 years [15–31]. In 12 studies, 54% to 100% of patients were white [15–17, 19–21, 23–26, 29, 31]. A majority of enrolled patients were Asian in two studies (54% [27] and 100% [30]). Two studies reported nearly equivalent proportions of white and Asian patients [22, 32]. Most studies (*n* = 11) were conducted in an international setting, enrolling patients in multiple countries and continents. Three studies were conducted solely in the United States (US) [17, 24, 31], while others enrolled patients in both the US and Japan [27], Europe [16], France [18], or China [30].

**Table 1:** Study and patient characteristics by anti-HER2 drugs

Citation	Trial no.; alias	Country	Trial phase	Date range of patient enrollment	Drug^a^	Sample size, *n*	Age, median (range), y	White,%	Asian, %	HR+, %	Months since initial Dx, median	Previous treatments		
												Chemo-therapy,%	Radiotherapy,%	Lines of systemic therapy, median (range)^b^
**Trastuzumab**														
Infante et al. (2009) [24]	NR	US	2	2001–2007	**Trastuzumab** + vinorelbine + docetaxel + dexamethasone	60	53 (28–79)	86	NR	42	NR	38	NR	NR
Hurvitz et al. (2013) [23]	NR; J2101	Belgium France Netherlands Spain US	2	2009–2012	**Trastuzumab** + paclitaxel + everolimus	55	56 (31–83)	87.3	0	NR	NR	100	NR	NR
Andre et al. (2014) [19]	NCT01007942; BOLERO-3	21 countries from Asia–Pacific Europe Middle East North America South America	3	2009–2012	**Trastuzumab** + vinorelbine + everolimus	284	54.5 (30–81)	69	24	ER + : 53 PR + : 37	NR	99	100	1 (0 to ≥ 4)
					**Trastuzumab** + vinorelbine + placebo	285	54 (25–77)	69	22	ER + : 53 PR + : 37	NR	100	100	1 (0 to ≥ 4)
Hurvitz et al. (2015) [22]	NCT00876395; BOLERO-1	28 countries from Africa Asia–Pacific Europe Middle East North America South America	3	2009–2012	**Trastuzumab** + paclitaxel + everolimus	472	54 (23–86)	45	41	57	NR	45	36	NR
					**Trastuzumab** + paclitaxel + placebo	238	52 (19–82)	41	44	57	NR	51	41	NR
Acevedo-Gadea et al. (2015) [17]	NR	US	2	2007–2010	**Trastuzumab** + sirolimus	11	57 (38–62)	72.7	NR	45.4	NR	100	NR	3 (2 to > 3)
Harbeck et al. (2016) [32]	NCT01125566	41 countries from Africa Asia–Pacific Europe Middle East North America South America	3	2010–2013	**Trastuzumab** + vinorelbine	169	53.1^c^ (NR)	43	48	ER + : 48 PR + : 30	22	100	NR	NR
Ajgal et al. (2017) [18]	NR	France	Retrospective study	2013–2015	**Trastuzumab** + **pertuzumab** + docetaxel (+ radiotherapy)	23	47 (33–85)	NR	NR	52	NR	30	30	NR
Loi et al. (2019) [28]^d^	NCT02129556; PANACEA	Australia Austria Belgium France Italy	2	2015–2017	**Trastuzumab** + pembrolizumab	PD-L1 + : 40 PD-L1-: 12	PD-L1 + : 49 (28–72) PD-L1-: 57 (43–61)	NR	NR	PD-L1 + : 43 PD-L1-: 50	PD-L1 + : 40.0^e^ PD-L1-: 71.5^e^	PD-L1 + : 100 PD-L1-: 100	NR	NR
**Lapatinib**														
Blackwell et al. (2009) [31]	NR	US	2	2002–2005	**Lapatinib**	78	54.5 (26–79)	76	NR	54	NR	96	59	2 (1 to ≥ 5)
Jagiello-Gruszfeld et al. (2010) [25]	NCT00356811, EGF105764	Latvia Poland Romania Russia	2	2006–2007	**Lapatinib** + paclitaxel	57	52 (32–69)	100	0	42	NR	58	47	All patients had no previous treatment for metastatic disease
Capri et al. (2010) [20]	NR; LEAP	45 countries from Asia–Pacific Europe North America South America	Open-label, expanded access program	2006–2008	**Lapatinib** + capecitabine	4,283	52 (21–86)	82.3	14.7	NR	NR	100	NR	NR
Xu et al. (2011) [30]	NCT00508274, EGF109491	China	3	Up to 2008	**Lapatinib** + capecitabine	52	50 (26–71)	0	100	48.1	NR	100	NR	2 (0 to ≥ 3)
**T-DM1**														
Dieras et al. (2014) [21]	Data pooled from 6 studies and an open-label extension study^f^	Countries from Asia–Pacific Europe Middle East North America South America	1, 2, 3^f^	Up to 2012	**T-DM1**	884	53 (25–85)	78.3	11.2	53.8	NR	≥ 91	NR	5 (0–19)
Krop et al. (2014) [26]	NCT01419197; TH3RESA	22 countries from Asia–Pacific Europe North America South America	3	2011–2013	**T-DM1**	404	53 (27–89)	80	14	51	NR	100	NR	4 (1–14)^g^
Montemurro et al. (2019) [29]	NCT01702571; KAMILLA	40 countries from Asia–Pacific Europe Middle East North America South America	3	2012–2014	**T-DM1**	2,002	55 (26–88)	69.8	3.6	61.5	60	100	NR	2 (0 to ≥ 4)
**T-DXd**														
Tamura et al. (2019) [27]	NCT02564900	Japan US	1	2015–2018	**T-DXd**	115	55 (47–66)^g^	NR	≥ 54	70	69.7^d^	100	82	7^h^ (5–11)^g^
Modi et al. (2019) [15]	NCT03248492; DESTINY-Breast01	8 countries from Europe Asia North America	2	2017–2018	T-**DXd**	184	55 (28–96)	54.9	38.0	52.7	NR	100	NR	6 (2–27)
**Trastuzumab duocarmazine**														
Banerji et al. (2019) [16]	NCT02277717	Belgium Netherlands UK Spain^i^	1	2014–2018	**Trastuzumab duocarmazine** (dose escalation)	39^j^	55 (47–63)	97	NR	NR	67	NR	NR	6 (2–8)^g^
					**Trastuzumab duocarmazine** (dose expansion)	146^j^	57 (49–65)	96	NR	NR	53	NR	NR	4 (3–7)^g^

*Dx* diagnosis, *ER +* estrogen receptor positive, *HER2* human epidermal growth factor receptor 2, *HR +* hormone receptor positive, *No.* number, *NR* not reported, *PD-L1* programmed cell death-1 ligand-1, *PR +* progesterone receptor positive, *T-DM1* trastuzumab emtansine, *T-DXd* trastuzumab deruxtecan, *UK* United Kingdom, *US* United States

^a^Anti-HER2 therapies are in bold

^b^Systemic therapy for advanced or metastatic disease, unless otherwise indicated

^c^Represents the mean instead of the median

^d^The sample was stratified by PD-L1 status

^e^Time since diagnosis of metastatic breast cancer

^f^Six trials: (1) NCT00829166, BO21977; EMILIA (phase 3), (2) NCT00679341, BO21976 (phase 2), (3) NCT00679211 (phase 2), (4) NCT00509769 (phase 2), (5) NCT00943670 (phase 2), and (6) NCT00932373 (phase 1). Open-label extension (phase 2): NCT00781612, BO25430

^g^Interquartile range

^h^Includes hormone therapies for breast cancer and treatments received in the (neo)adjuvant setting

^i^ atients recruited from hospitals in Spain were enrolled only during the dose-expansion phase

^j^The dose-escalation phase and dose-expansion phase included patients with HER2 + metastatic breast cancer (dose expansion: *n* = 50 [34.2%]) as well as patients with HER2-low (i.e., low or no expression of HER2) metastatic breast cancer (dose expansion: *n* = 49 [33.6%]) and other nonbreast solid tumors (dose expansion: gastric cancer, *n* = 17 [11.6%]; urothelial cancer, *n* = 16 [11.0%]; endometrial cancer, *n* = 14 [9.6%])

The reported proportion of patients with positive hormone receptor (i.e., estrogen receptor and/or progesterone receptor) status ranged from 42 to 70%. Nearly all patients had an Eastern Cooperative Oncology Group (ECOG)/World Health Organization performance status of 0 or 1 (at least 92% in each treatment group); however, five studies enrolled a minimal number of patients with an ECOG performance status of 2, ranging from less than 1% to 8% of patients [15, 19, 24, 26, 29]. The shortest time (median) since breast cancer diagnosis was 22 months [32] and the longest was 71.5 months (among patients negative for programmed cell death-1 ligand-1 [PD-L1]) [28]. All but one study [25] included patients who had received previous breast cancer treatment. Among the six studies that reported on radiotherapy history [18, 19, 22, 25, 27, 31], the proportion of patients with previous radiotherapy ranged from 30% [18] to 100% [19]. The reported previous lines (median) of systemic therapy for advanced or metastatic disease ranged from one line [19] to five or more lines of therapy [15, 21, 27], with 2–4 previous lines of therapy being the most commonly reported [16, 17, 26, 29–31].

Articles in this review reported on adverse drug reactions for currently approved or investigational anti-HER2 therapies. The approved therapies include trastuzumab combination therapy, T-DM1, T-DXd (approved in the US), and lapatinib combination therapy. Trastuzumab duocarmazine was the only investigational therapy. Patient enrollment was conducted during 2001–2017 for trastuzumab studies, up to 2008 for lapatinib studies, up to 2014 for T-DM1 studies, 2015–2018 for T-DXd studies, and 2014–2018 for the trastuzumab duocarmazine study.

### Drug-induced ILD incidence and severity

A summary of drug-induced ILD incidence and severity reported in each study is presented in Table 2. A summary of drug-induced ILD incidence and severity by anti-HER2 therapy, including trastuzumab, lapatinib, T-DM1, T-DXd, and trastuzumab duocarmazine is provided in Table S-2 (Supplementary Material).

**Table 2:** Summary of the incidence and severity of drug-induced ILD in patients with HER2-positive metastatic breast cancer who received anti-HER2 therapy

Citation	Drug^a^	Sample size	Reported ILD conditions						Current line of treatment for advanced disease^b^	Median treatment duration
			Condition	Cases, *n* (%)	Grade 1–2, *n* (%)	Grade 3, *n* (%)	Grade 4, *n* (%)	Grade 5 (death), *n* (%)		
**Trastuzumab**										
Infante et al. (2009 [24]	**Trastuzumab** + vinorelbine + docetaxel + dexamethasone	60	All ILD conditions	2 (3.3)	0 (0.0)	2^c^ (3.3)		0 (0.0)	First line	11 mo (range 1–22 mo)
			Pneumonitis	1 (1.7)	0 (0.0)	1^c^ (1.7)		0 (0.0)		
			Respiratory distress	1 (1.7)	0 (0.0)	1^c^ (1.7)		0 (0.0)		
Hurvitz et al. (2013) [23]	**Trastuzumab** + paclitaxel + everolimus	55	Pneumonitis	4 (7.3)	2 (3.6)	2 (3.6)	0 (0.0)	0 (0.0)	Later line	Trastuzumab and everolimus: 24 weeks Paclitaxel: 21.7 weeks
Andre et al. (2014) [19]	**Trastuzumab** + vinorelbine + everolimus	284	All ILD conditions	26 (9.2)	20 (7.0)	4 (1.4)	2 (0.7)	0 (0.0)	First line: 16%	Trastuzumab: 25.1 weeks (range 1.0–169.7 weeks) Vinorelbine: 24.0 weeks (range 1.0–169.7 weeks) Everolimus: 24.8 weeks (range 0.9–169.3) weeks
			Pneumonitis	16 (5.6)	13 (4.6)	1 (0.4)	2 (0.7)	0 (0.0)	Later line: 84%	
			ILD	10 (3.5)	7 (2.5)	3 (1.1)	0 (0.0)	0 (0.0)		
	**Trastuzumab** + vinorelbine + placebo	285	All ILD conditions	11 (3.9)	6 (2.1)	4 (1.4)	1 (0.4)	0 (0.0)	First line: 16%	Trastuzumab: 24.0 weeks (range 1.0–138.0 weeks) Vinorelbine: 23.1 weeks (range 1.0–137.0 weeks) Placebo: 22.9 weeks (range 0.1–140.6 weeks)
			Pneumonitis	9 (3.2)	4 (1.4)	4 (1.4)	1 (0.4)	0 (0.0)	Later line: 84%	
			ILD	2 (0.7)	2 (0.7)	0 (0.0)	0 (0.0)	0 (0.0)		
Hurvitz et al. (2015) [22]	**Trastuzumab** + paclitaxel + everolimus	472	All ILD conditions	101 (21.4)	67 (14.2)	27 (5.7)	4 (0.8)	3 (0.6)	Later line	NR
			Pneumonitis	80 (16.9)	54 (11.4)	19 (4.0)	4 (0.8)	3 (0.6)		
			ILD	21 (4.4)	13 (2.8)	8 (1.7)	0 (0.0)	0 (0.0)		
	**Trastuzumab** + paclitaxel + placebo	238	All ILD conditions	11 (4.6)	10 (4.2)	0 (0.0)	1 (0.4)	0 (0.0)	Later line	NR
			Pneumonitis	10 (4.2)	9 (3.8)	0 (0.0)	1 (0.4)	0 (0.0)		
			ILD	1 (0.4)	1 (100)	0 (0.0)	0 (0.0)	0 (0.0)		
Acevedo-Gadea et al. (2015) [17]	**Trastuzumab** + sirolimus	11	Pneumonitis	1 (9.1)	0 (0.0)	1 (9.1)	0 (0.0)	0 (0.0)	Later line	Range: 3–58 weeks
Harbeck et al. (2016) [32]	**Trastuzumab** + vinorelbine	169	All ILD conditions	0 (0.0)	NA	NA	m	NA	First line: 42%	4.7 mo (range 2.1–7.4 mo)
			Pneumonitis	0 (0.0)					Second line: 58%	
			ILD	0 (0.0)						
Ajgal et al. (2017) [18]	**Trastuzumab** + **pertuzumab** + docetaxel (+ radiotherapy)	23	Pneumonitis (radiation)	2 (8.7)	2 (8.7)	0 (0.0)	0 (0.0)	0 (0.0)	First line	Trastuzumab: 12.4 mo (range 5.8–21.6 mo) Pertuzumab: 11.3 mo (range 3.5–21.6 mo)
Loi et al. (2019) [28]	**Trastuzumab** + pembrolizumab	52	Pneumonitis	4 (7.7)	2 (3.8)	1 (1.9)	1 (1.9)	0 (0.0)	Later line	NR
**Lapatinib**										
Blackwell et al. (2009) [31]	**Lapatinib**	78	Interstitial pneumonitis	0 (0.0)	0 (0.0)	0 (0.0)	0 (0.0)	0 (0.0)	Later line	8.4 weeks (range 1–70 weeks)
Jagiello-Gruszfeld et al. (2010) [25]	**Lapatinib** + paclitaxel	57	Pulmonary fibrosis	1 (1.8)	NR	NR	NR	0 (0.0)	First line	Lapatinib: 45 weeks (range 2–87 weeks)
Capri et al. (2010) [20]	**Lapatinib** + capecitabine	4283	All ILD conditions	7 (0.2)	NR	NR	NR	0 (0.0)	Later line	24.7 weeks (maximum, 131.3 weeks)
			Pneumonitis	3 (0.1)						
			ILD	2 (< 0.1)						
Xu et al. (2011) [30]	**Lapatinib** + capecitabine	52	Interstitial pneumonitis	0 (0.0)	NA	NA	NA	NA	Later line	Lapatinib: 24.3 weeks Capecitabine: 16 weeks
**T-DM1**										
Dieras et al. (2014) [21]	**T-DM1**	884	Pneumonitis	10 (1.1)	7 (0.8)	1 (0.1)	1 (0.1)	1 (0.1)	First line: 18% Later line: 82%	6.3 mo (range 0–53.4 mo)
Krop et al. (2014) [26]	**T-DM1**	404	Pneumonitis	1 (0.2)	0 (0.0)	0 (0.0)	0 (0.0)	1 (0.2)	Later line	4.24 mo (IQR, 2.23–6.24 mo)
Montemurro et al. (2019) [29]	**T-DM1**	2002	All ILD conditions	4 (0.2)	0 (0.0)	0 (0.0)	0 (0.0)		First or second line: 30% ≥ Third line: 66% Missing^d^: 4%	5.6 mo (range 0–46 mo)
			Pneumonitis	2 (0.1)	0 (0.0)	0 (0.0)	0 (0.0)			
			ILD	1 (< 0.1)	0 (0.0)	0 (0.0)	0 (0.0)			
			Pulmonary fibrosis	1 (< 0.1)	0 (0.0)	0 (0.0)	0 (0.0)			
**T-DXd**										
Tamura et al. (2019) [27]	**T-DXd**	115	All ILD conditions	20 (17.4)	17 (14.8)	1 (0.9)	0 (0.0)	2 (1.7)	Later line	8.3 mo (IQR, 4.4–12.0 mo)
			Pneumonitis	8 (7.0)	6 (5.2)	0 (0.0)	0 (0.0)	2 (1.7)		
			ILD	6 (5.2)	5 (4.3)	1 (0.9)	0 (0.0)	0 (0.0)		
			Organizing pneumonia	6 (5.2)	6 (5.2)	0 (0.0)	0 (0.0)	0 (0.0)		
Modi et al. (2019) [15]	**T-DXd**	184	All ILD conditions^e^	25 (13.6)	20 (10.9)	1 (0.5)	0 (0.0)	4^f^ (2.2)	Later line	10.0 mo (range 0.7–20.5 mo)
**Trastuzumab duocarmazine**										
Banerji et al. (2019) [16]	**Trastuzumab duocarmazine** (dose-escalation phase)	39^g^	Pneumonitis	3 (7.7)	3 (7.7)	0 (0.0)	0 (0.0)	0 (0.0)	Later line	3.5 mo (IQR, 1.4–5.4 mo)
	**Trastuzumab duocarmazine** (dose-expansion phase)	146^g^	Pneumonitis	1 (0.7)	0 (0.0)	0 (0.0)	0 (0.0)	1 (0.7)		

*CTCAE* common toxicity criteria for adverse events, *HER2* human epidermal growth factor receptor 2, *ICH* International Council for Harmonisation, *ILD* interstitial lung disease, *IQR* interquartile range, *NA* not applicable, *NCI* National Cancer Institute, *NR* not reported, *T-DM1* trastuzumab emtansine, *T-DXd* trastuzumab deruxtecan

^a^Anti-HER2 therapies are in bold

^b^First-line therapy includes patients who have previously received no systemic therapy for advanced or metastatic disease or have received only neo-adjuvant or adjuvant therapy; second-line therapy includes patients who are indicated to have previously received first-line therapy but not later lines; later line includes patients who are indicated to have received previous systemic therapy for advanced or metastatic disease and may include second-line therapy and/or later line therapy

^c^The authors describe these events as “serious pulmonary events,” and therefore it is likely that they may be either grade 3 or 4 when considering the ICH's E2A guidelines [39] and the NCI’s CTCAE, v5.0 [40]

^d^Missing information on previous treatment lines in the metastatic setting

^e^Treatment-related ILD events were confirmed by an independent adjudication committee

^f^Deaths were attributed to ILD by independent adjudication and were initially reported as respiratory failure (*n* = 1), acute respiratory failure (*n* = 1), lymphangitis (*n* = 1), or pneumonitis (*n* = 1)

^g^The dose-escalation phase and dose-expansion phase included patients with HER2 + metastatic breast cancer (dose expansion: *n* = 50 [34.2%]) as well as patients with HER2-low (i.e., low or no expression of HER2) metastatic breast cancer (dose expansion: *n* = 49 [33.6%]) and other nonbreast solid tumors (dose expansion: gastric cancer, *n* = 17 [11.6%]; urothelial cancer, *n* = 16 [11.0%]; endometrial cancer, *n* = 14 [9.6%])

#### Trastuzumab

Eight studies reported incidence and severity of drug-induced ILD in a total of 1,642 patients receiving trastuzumab therapy; of these patients, 162 (9.9%) had a reported ILD event. Overall, there were 3 (0.2%) ILD-related deaths among those receiving trastuzumab therapy. Two trastuzumab studies reported at least one case of specifically ILD [19, 22]. Seven trastuzumab studies reported at least one case of pneumonitis [17–19, 22–24, 28], of which six studies reported at least one case of grade 3 or higher pneumonitis and one study reported grade 1–2 pneumonitis cases related to radiotherapy [18]. Four studies assessed patients who received trastuzumab combined with an mTOR inhibitor, including everolimus [19, 22, 23] and sirolimus [17]. One study reported an ILD incidence of 9.2% (*n* = 26) among patients who received trastuzumab for 25.1 weeks (median) combined with vinorelbine and everolimus; the ILD incidence was lower in the comparator arm (*n* = 11 [3.9%]), which received trastuzumab for 24.0 weeks (median) combined with vinorelbine and placebo instead of everolimus; however, compared with the everolimus arm, a similar proportion of patients in the comparator arm (*n* = 5 [1.8%]) were diagnosed with grade 3 or 4 ILD [19]. Other studies with patients receiving trastuzumab combined with vinorelbine, either alone for 4.7 months (median) [32] or combined with docetaxel (a taxane chemotherapeutic agent) and dexamethasone (a corticosteroid) for 11 months (median) [24], reported a lower ILD incidence (*n* = 0 [0%] and *n* = 2 [3.3%], respectively) than studies with patients who received trastuzumab combined with an mTOR inhibitor.

#### Lapatinib

Four studies reported on a total of 4,470 patients who received lapatinib, a dual epidermal growth factor receptor/HER2 tyrosine kinase inhibitor; eight of these patients (0.2%) had at least one reported ILD event. The earliest of these studies reported no ILD cases among patients who received lapatinib for 8.4 weeks (median) [31]. An expanded access study conducted in 45 countries (sample size, *n* = 4,283) [20] and a phase 3 study conducted in China (sample size, *n* = 52) [30] reported an ILD incidence of 0.2% (*n* = 7) and 0.0% (no cases), respectively, among patients who received lapatinib, combined with capecitabine, for 24.7 and 24.3 weeks (median), respectively. Among all studies that assessed lapatinib-based therapies, the highest ILD incidence (1.8%, consisting of one case of pulmonary fibrosis) was reported in a phase 2 study (sample size, *n* = 57) conducted in eastern Europe among patients who received lapatinib combined with paclitaxel for 45 weeks (median) [25].

#### Trastuzumab emtansine

Three studies reported on 3,290 patients who received T-DM1, an antibody–drug conjugate that contains the trastuzumab monoclonal antibody bound to the cytotoxic agent DM1, with a treatment duration ranging from 4.2 to 6.3 months (median) [21, 26, 29]. Across these studies, 15 patients (0.5%) had a reported ILD event, and 6 patients (0.2%) had an ILD-related death. Dieras et al. (sample size, *n* = 884) reported the highest ILD incidence (pneumonitis, *n* = 10 [1.1%]), including 2 patients (0.2%) with grade 3–4 pneumonitis and one pneumonitis-related death (0.1%) [21]. Both Krop et al. (sample size, *n* = 404) and Montemurro et al. (sample size, *n* = 2,002) only reported deaths related to ILD, both with an incidence of 0.2% (*n* = 1 and *n* = 4, respectively) [26, 29].

#### Trastuzumab deruxtecan

T-DXd is a HER2-targeted antibody–drug conjugate composed of a humanized monoclonal antibody with the same amino acid sequence as trastuzumab, a cleavable tetrapeptide-based linker, and a potent topoisomerase I inhibitor payload [33–35]. A phase 1 study enrolled 115 patients (dose-expansion phase) from Japan (*n* = 62 [54%]) and the US (*n* = 53 [46%]) who received T-DXd for 8.3 months (median) and reported an ILD incidence of 17.4% (*n* = 20; grade 1–2, *n* = 17 [incidence, 14.8%]; grade 3, *n* = 1 [incidence, 0.9%]; no grade 4; deaths, *n* = 2 [incidence, 1.7%]), including cases of pneumonitis (*n* = 8), specifically ILD (*n* = 6), and organizing pneumonia (*n* = 6) [27]. The phase 2 study assessed 184 patients enrolled from North America (*n* = 53 [28.8%]), Asia (*n* = 63 [34.2%]), and Europe (*n* = *n* = 68 [37.0%]) who received T-DXd for 10.0 months (median) [15]. Twenty-five patients (13.6%) had a treatment-related ILD event, most of which were grade 1–2 (*n* = 20 [incidence, 10.9%]), one grade 3 (incidence, 0.5%), no grade 4, and four ILD-related deaths (incidence, 2.2%).

#### Trastuzumab duocarmazine

Trastuzumab duocarmazine is a HER2-targeted antibody–drug conjugate composed of trastuzumab bound to a linker drug containing duocarmycin. One phase 1 study reported on ILD among patients with either MBC (*n* = 99 [67.8%] in the dose-expansion phase), gastric cancer, urothelial cancer, or endometrial cancer and receiving trastuzumab duocarmazine for 3.5 months (median) [16]. The study included a dose-escalation phase (sample size, *n* = 39) and a dose-expansion phase (sample size, *n* = 146) and reported three cases of pneumonitis (7.7%, all grade 1–2) in the dose-escalation phase and one pneumonitis-related death (0.7%) in the dose-expansion phase.

### ILD severity by line of therapy for advanced disease

Across all studies, the incidence of any-grade ILD was similar between patients who received an anti-HER2 drug as first-line therapy (3.6% [*n* = 5]) versus later line therapy (2.3% [*n* = 229]) (Table S-3, Supplementary Material). While studies reporting on patients who received an anti-HER2 drug as first-line therapy reported no ILD-related deaths, studies assessing later line therapy reported a 0.2% incidence of ILD-related death (*n* = 16, including ILD [*n* = 5 (< 0.1%)], pneumonitis [*n* = 10 (0.1%)], and pulmonary fibrosis [*n* = 1 (< 0.1%)]); however, among the studies included in this review, more patients received anti-HER2 therapy as later line therapy (*n* = 9,749) than as first-line therapy (*n* = 140).

Among patients who received an anti-HER2 drug as first-line therapy for advanced MBC, the ILD incidence was 1.8% (*n* = 1) [25], 3.3% (*n* = 2) [24], and 8.7% (*n* = 2) [18] across the three studies reporting on patients receiving lapatinib-based therapy for 10.4 months (median), trastuzumab-based therapy for 11 months (median), or trastuzumab-based therapy for 12.4 months (median) combined with pertuzumab for 11.3 months (median) and radiotherapy, respectively. In studies reporting only on patients who previously received systemic therapy for advanced MBC, the ILD incidence was as low as 0% among patients receiving lapatinib alone for 1.9 months (median) as later line therapy [31] and as high as 21.4% (*n* = 101) among patients receiving trastuzumab combined with paclitaxel and everolimus as later line therapy (treatment duration not reported) [22]. Among patients who received T-DM1 for 4.2 months (median) as later line therapy, only one case (0.2%) of pneumonitis was reported, which resulted in death [26]. Four studies reported on patients receiving either first-line or later line therapy for advanced or metastatic disease, with the majority of patients receiving later line therapy (range 58–84%) across the studies [19, 21, 29, 32].

### Monitoring and management of ILD

None of the studies in this review reported on specific guidelines for monitoring for ILD before an ILD diagnosis among patients receiving anti-HER2 therapies. However, 10 of the 18 studies (55.6%) indicated that ILD-related events were managed via dose interruption, dose reduction, or treatment discontinuation [15–17, 22, 23, 25–28, 30]. The most common approaches included dose interruption and reduction for grades 2 and 3 and treatment discontinuation (often until the improvement of the ILD condition) for grades 3 and 4.

Two studies in this review provided detailed guidelines for management of drug-induced ILD. In a phase 2 clinical trial of T-DXd, Modi et al. [15] recommend T-DXd dose interruption and possible systemic steroids for grade 1 events, and permanent T-DXd discontinuation with prompt initiation of systemic steroids for grade 2, 3, or 4 events; hospitalization is required for grade 3 or 4 events. In contrast, in a phase 3 clinical trial of trastuzumab combined with paclitaxel and either everolimus or placebo, Hurvitz et al. [22] recommend no specific treatment for grade 1 events, everolimus dose reduction and paclitaxel dose interruption for grade 2 events, additional dose interruption of everolimus and trastuzumab for grade 3 events, and treatment discontinuation for grade 4 events; corticosteroids are recommended for grades 2, 3, or 4 events.

## Discussion

Drug-induced ILD has been reported among patients with HER2-positive MBC receiving anti-HER2 therapies, including trastuzumab, lapatinib, T-DM1, T-DXd, and trastuzumab duocarmazine. The highest ILD incidence was reported in patients who received trastuzumab combined with an mTOR inhibitor (everolimus [range 7.3–21.4%] or sirolimus [9.1%]), and in patients who received T-DXd (range 13.6–17.4%). The incidence of ILD-related deaths was highest among patients receiving T-DXd (range 1.7–2.2%), whereas in patients receiving trastuzumab, lapatinib, T-DM1, or trastuzumab duocarmazine, the incidence was lower (range 0.1–0.6%). The elevated incidence of drug-induced ILD among those who received an mTOR inhibitor combined with trastuzumab is consistent with previous studies demonstrating associations between mTOR inhibitors and ILD among cancer patients [10, 36]. The lowest incidence of drug-induced ILD was among patients who received lapatinib-based therapy (range 0–1.8%) or T-DM1 (range 0.2–1.1%).

Disease management guidelines for drug-induced ILD were provided in two studies in this review: one on managing ILD induced by T-DXd [15] and the other on ILD induced by trastuzumab combined with paclitaxel and everolimus [22]. The guidance provided by these two studies differs, as they were conducted in the context of two different anti-HER2 treatment regimens. The guidelines for trastuzumab in combination with paclitaxel and everolimus are consistent with the pneumonitis management guidance provided in the everolimus prescribing information [37], both recommending everolimus dose reduction and/or interruption for grade 2 or 3 events, but no change in treatment regimen for grade 1. The guidelines for T-DXd-induced ILD recommend T-DXd dose interruption for grade 1 and permanent T-DXd treatment discontinuation for patients with grade 2, 3, or 4, which is similar to the management recommendations for drug-induced lung injuries in Kubo et al. [13] and for pneumonitis induced by immune checkpoint inhibitors in the American Society of Clinical Oncology Practice Guideline [38]. The clinical significance of an ILD diagnosis together with the relatively high incidence of ILD induced by some anti-HER2 therapies highlights the need for consensus in ILD monitoring and management guidelines in the context of anticancer treatment for metastatic disease.

There are limitations associated with this review. The search was limited to the past 10 years and to English-language articles only. Additionally, studies reporting on ILD using a diagnosis term other than those prespecified in the inclusion criteria may have been excluded. Lastly, there was no quality assessment of the included studies (e.g., respiratory failure). However, a quality assessment of included studies was not necessary to accomplish the primary study objective of reporting on the incidence of ILD as an adverse drug reaction in the context of a clinical trial or observational study.

## Conclusions

ILD is a well-described adverse drug reaction associated with anti-HER2 therapies, with the highest ILD incidence reported among patients receiving trastuzumab and everolimus combination therapy. Drug-induced ILD is typically managed via dose reduction, dose interruption, or treatment discontinuation; however, detailed ILD management guidelines are available for only two anti-HER2 treatment regimens (i.e., T-DXd and trastuzumab combined with paclitaxel and either everolimus or placebo), necessitating the development of standard guidelines across all anti-HER2 therapies for MBC. The dearth of published ILD monitoring approaches in the context of HER2-positive MBC and anti-HER2 therapy highlights the need to identify risk factors and the underlying etiology of ILD to develop effective strategies for monitoring for ILD among these patients.

## Electronic supplementary material

Below is the link to the electronic supplementary material.

Supplementary file1 (DOCX 29 kb)

## Data Availability

All data generated or analyzed during this study are included in this published article and its supplementary information files.
