# Supplementary material for: Incidence of pneumonitis/interstitial lung disease induced by HER2-targeting therapy for HER2-positive metastatic breast cancer
Source: Breast Cancer Res Treat. 2020 Jun 26;183(1):23–39. doi: 10.1007/s10549-020-05754-8 (PMC7376509; doi:10.1007/s10549-020-05754-8)
Supplement: Supplementary file 1 — Supplementary file1 (DOCX 29 kb) [file 10549_2020_5754_MOESM1_ESM.docx]

# Supplementary Material

Table S-1. Inclusion and Exclusion Criteria for Level 1 (Titles and Abstracts) and Level 2 (Full-Text) Screening

| Criterion | Included | Excluded |
| --- | --- | --- |
| Population | Female adults (aged 18 years and over)  Patients diagnosed with metastatic, recurrent, advanced, incurable, or unresectable HER2 positive breast cancer (stages 3-4)  Taking a pharmacological anticancer agent | Males  Aged under 18 years  Patients with HER2-negative or stage 1 breast cancer |
| Interventions | Currently approved anti-HER2 therapies:   - Trastuzumab (Herceptin) - Pertuzumab (Perjeta, Omnitarg) - Trastuzumab emtansine (T-DM1, Kadcyla) - Lapatinib (Tykerb)   Investigational anti-HER2 therapies:   - Trastuzumab deruxtecan (T-DXd, DS-8201a) - Trastuzumab-dkst (Ogivri) - Trastuzumab-dttb (Ontruzant) - Trastuzumab-pkrb (Herzuma) - Trastuzumab-qyyp (Trazimera) - Trastuzumab duocarmazine (SYD985) - Neratinib (Nerlynx) (currently approved for early stage disease) - Margetuximab (MGAH22, a monoclonal antibody) - Pyrotinib - Tucatinib - MM-302 (an antibody-drug conjugate) | Nonpharmacological studies  Patients receiving surgical or radiation intervention in place of chemotherapy  Patients receiving CDK4/6 inhibitors |
| Comparators | Any (i.e., placebo, approved agent for HER2+ metastatic breast cancer, or investigational agent for HER2 positive metastatic breast cancer) | None |
| Outcomes | ILD   - Pulmonary fibrosis - Pneumonitis - Organizing pneumonia - Acute interstitial pneumonitis - Diffuse parenchymal lung disease - Pulmonary eosinophilia - Interstitial lung disease | Trials or studies not reporting ILD |
| Study design | Randomized controlled trials   - Parallel and crossover designs - Placebo and active control - No minimum trial size - No minimum trial duration - Phases 2, 3, and 4   Single-arm studies  Observational research studies (e.g., prospective cohort study, retrospective database study, cohort study, case-control study)  Literature reviews and meta-analyses^a^ | Natural history studies  Consensus reports  Phase 1 trials^b^  Preclinical studies  Nonsystematic reviews  Case reports  Case studies/series  Editorials  Commentaries  Letters  Guideline or position statements  Economic analyses  Animal or other nonhuman (e.g., bench) studies |

CDK = cyclin-dependent kinase; HER2 = human epidermal growth factor receptor 2; ILD = interstitial lung disease; T-DM1 =  trastuzumab emtansine.

^a^ Literature reviews and meta-analyses will not be included in their own right but will be used to identify primary studies not previously identified.

^b^ Phase 1 studies of T-DXd and trastuzumab duocarmazine were included in the review as ILD is anticipated to be a serious drug-induced adverse event for these agents.

Table S-2. Summary of ILD-Related Events and Death in Patients Who Received Anti-HER2 Drugs: All Patient Populations and Settings

| Drug | Total Patients, n | Patients With ILD Conditions | | |
| --- | --- | --- | --- | --- |
|  |  | ILD Condition | Cases, n (%) | Deaths, n (%) |
| Trastuzumab |  |  |  |  |
| All combinations | 1,642 | All ILD conditions | 162 (9.9) | 3 (0.2) |
|  |  | ILD | 34 (2.1) | 0 (0.0) |
|  |  | Pneumonitis | 127 (7.7) | 3 (0.2) |
|  |  | Respiratory distress | 1 (<0.1) | 0 (0.0) |
| Combined with an mTOR inhibitor^a^ | 818 | All ILD conditions | 132 (16.1) | 3 (0.4) |
|  |  | ILD | 31 (3.8) | 0 (0.0) |
|  |  | Pneumonitis | 101 (12.3) | 3 (0.4) |
| Combined with Pertuzumab | 23 | Radiation pneumonitis | 2 (8.7) | 0 (0.0) |
| Lapatinib | 4,470 | All ILD conditions | 8 (0.2) | 0 (0.0) |
|  |  | ILD | 2 (<0.1) | 0 (0.0) |
|  |  | Pneumonitis | 3 (0.1) | 0 (0.0) |
|  |  | Pulmonary fibrosis | 1 (<0.1) | 0 (0.0) |
|  |  | Lung infiltration | 2 (<0.1) | 0 (0.0) |
| T-DM1 | 3,290 | All ILD conditions | 15 (0.5) | 6 (0.2) |
|  |  | ILD | 1 (<0.1) | 1 (<0.1) |
|  |  | Pneumonitis | 13 (0.4) | 4 (0.1) |
|  |  | Pulmonary fibrosis | 1 (<0.1) | 1 (<0.1) |
| T-DXd | 299 | All ILD conditions | 45 (15.1) | 6 (2.0) |
|  |  | ILD | 31 (10.4) | 4 (1.3) |
|  |  | Pneumonitis | 8 (2.7) | 2 (0.7) |
|  |  | Organizing pneumonia | 6 (2.0) | 0 (0.0) |
| Trastuzumab duocarmazine | 185 | Pneumonitis | 4 (2.2) | 1 (0.5) |

HER2 = human epidermal growth factor receptor 2; ILD = interstitial lung disease; mTOR = mammalian target of rapamycin; T‑DM1 = trastuzumab emtansine; T‑DXd = trastuzumab deruxtecan.

^a^ Includes everolimus and sirolimus.

Table S-3. Summary of ILD-Related Events and Deaths by Line of Treatment for Advanced or Metastatic Breast Cancer in Patients Who Received Anti-HER2 Drugs

| Line of Treatment for Advanced Disease | Total Patients, n | Patients With ILD Conditions | | |
| --- | --- | --- | --- | --- |
|  |  | ILD Condition | Cases, n (%) | Deaths, n (%) |
| First line | 140 | All ILD conditions | 5 (3.6) | 0 (0.0) |
|  |  | Pneumonitis | 3 (2.1) | 0 (0.0) |
|  |  | Pulmonary fibrosis | 1 (0.7) | 0 (0.0) |
|  |  | Respiratory distress | 1 (0.7) | 0 (0.0) |
| Later line^a^ | 9,746 | All ILD conditions | 229 (2.3) | 16 (0.2) |
|  |  | ILD | 68 (0.7) | 5 (<0.1) |
|  |  | Pneumonitis | 152 (1.6) | 10 (0.1) |
|  |  | Pulmonary fibrosis | 1 (<0.1) | 1 (<0.1) |
|  |  | Organizing pneumonia | 6 (<0.1) | 0 (0.0) |
|  |  | Lung infiltration | 2 (<0.1) | 0 (0.0) |

ILD = interstitial lung disease.

^a^ Includes second line of treatment or later. Includes all studies where all or the majority of patients were receiving a later line of treatment.
